# Successful treatment of primary refractory DLBCL/HGBL - MYC/BCL2 transformed from FL using glofitamab: a case report

**DOI:** 10.3389/fimmu.2025.1566035

**Published:** 2025-03-31

**Authors:** Ming-qiang Chu, Ting-juan Zhang, Yuan Feng, Xun Shao, Yong-hui Ji, Jun Qian, Jing-dong Zhou

**Affiliations:** ^1^ Department of Hematology, The Affiliated People’s Hospital of Jiangsu University, Zhenjiang, Jiangsu, China; ^2^ Institute of Hematology, Jiangsu University, Zhenjiang, Jiangsu, China; ^3^ Zhenjiang Clinical Research Center of Hematology, Zhenjiang, Jiangsu, China; ^4^ The Key Lab of Precision Diagnosis and Treatment of Zhenjiang City, Zhenjiang, Jiangsu, China; ^5^ Department of Oncology, The Affiliated People’s Hospital of Jiangsu University, Zhenjiang, Jiangsu, China; ^6^ Department of Nuclear Medicine, The Affiliated People’s Hospital of Jiangsu University, Zhenjiang, Jiangsu, China

**Keywords:** DLBCL/HGBL-MYC/BCL2, transformed, primary refractory, glofitamab, case report

## Abstract

Diffuse large B-cell lymphoma/high-grade B-cell lymphoma with *MYC* and *BCL2* rearrangements (DLBCL/HGBL-*MYC*/*BCL2*) represents a distinct entity of mature aggressive B-cell lymphoma, constituting a substantial gap in the clinical management of DLBCL. Conventional R-CHOP-like chemoimmunotherapy regimens have demonstrated limited efficacy in DLBCL/HGBL-*MYC*/*BCL2*, and the clinical outcome remains poor, with a median overall survival of less than 2 years, and even shorter in cases transformed from indolent lymphoma. We reported a 66-year-old female was firstly diagnosed with follicular lymphoma, but presented with disease progression to DLBCL/HGBL-*MYC*/*BCL2* during the treatment with BR regimen. Moreover, the patient was also primary refractory to Pola-R-CHP. The patient achieved partial response following treatment with the CD20×CD3 bispecific antibody glofitamab and maintained long-term remission. Although only one successful case is presented, glofitamab could be considered as salvage therapy for transformed relapsed/refractory DLBCL/HGBL-*MYC*/*BCL2*.

## Introduction

Diffuse large B-cell lymphoma/high-grade B-cell lymphoma with *MYC* and *BCL2* rearrangements (DLBCL/HGBL-*MYC*/*BCL2*) represents a distinct entity of mature aggressive B-cell lymphoma, which is either *de novo* DLBCL or transformed from indolent lymphoma (1, 2). The efficacy of R-CHOP-like chemoimmunotherapy regimens in these patients has been demonstrated to be limited (3–5). The median overall survival (OS) of these patients is less than 2 years, shorter than in patients with single or no *MYC* rearrangements (6, 7). In the patients who have transformed from follicular lymphoma (FL), the median OS is only 7.9 months (8). The inferior clinical outcomes of these patients are attributed to distinctive cytomolecular genetics (9), with *MYC* and *BCL2* rearrangements having been revealed as pivotal contributors to the evolution of resistance (10). The median OS of the primary refractory patients was only 7.1 months (11). To address this dilemma, a range of treatment strategies are currently being investigated, including the dose-adjusted chemotherapy regimens, the incorporation of targeted agents, bispecific antibodies and chimeric antigen receptor T-cell (CAR-T). Few prospective trials have been reported for treating DLBCL/HGBL-*MYC*/*BCL2* patients. Although, in the ZUMA-12 trial, outcomes for patients with double-hit lymphoma were analyzed as a prespecified subgroup, showing high efficacy following axi-cel treatment, larger validation in ongoing phase 3 trials is critical given the limited subgroup size in this single-arm study (12, 13). Herein, we reported a case of DLBCL/HGBL-*MYC*/*BCL2* transformed from FL during the treatment with BR (bendamustine and rituximab) regimen, was primary refractory to Pola-R-CHP (polatuzumab vedotin, rituximab, cyclophosphamide, doxorubicin, and prednisone), and ultimately responded to the CD20×CD3 bispecific antibody glofitamab with a long-term partial response.

## Case report

**Figure 1:**
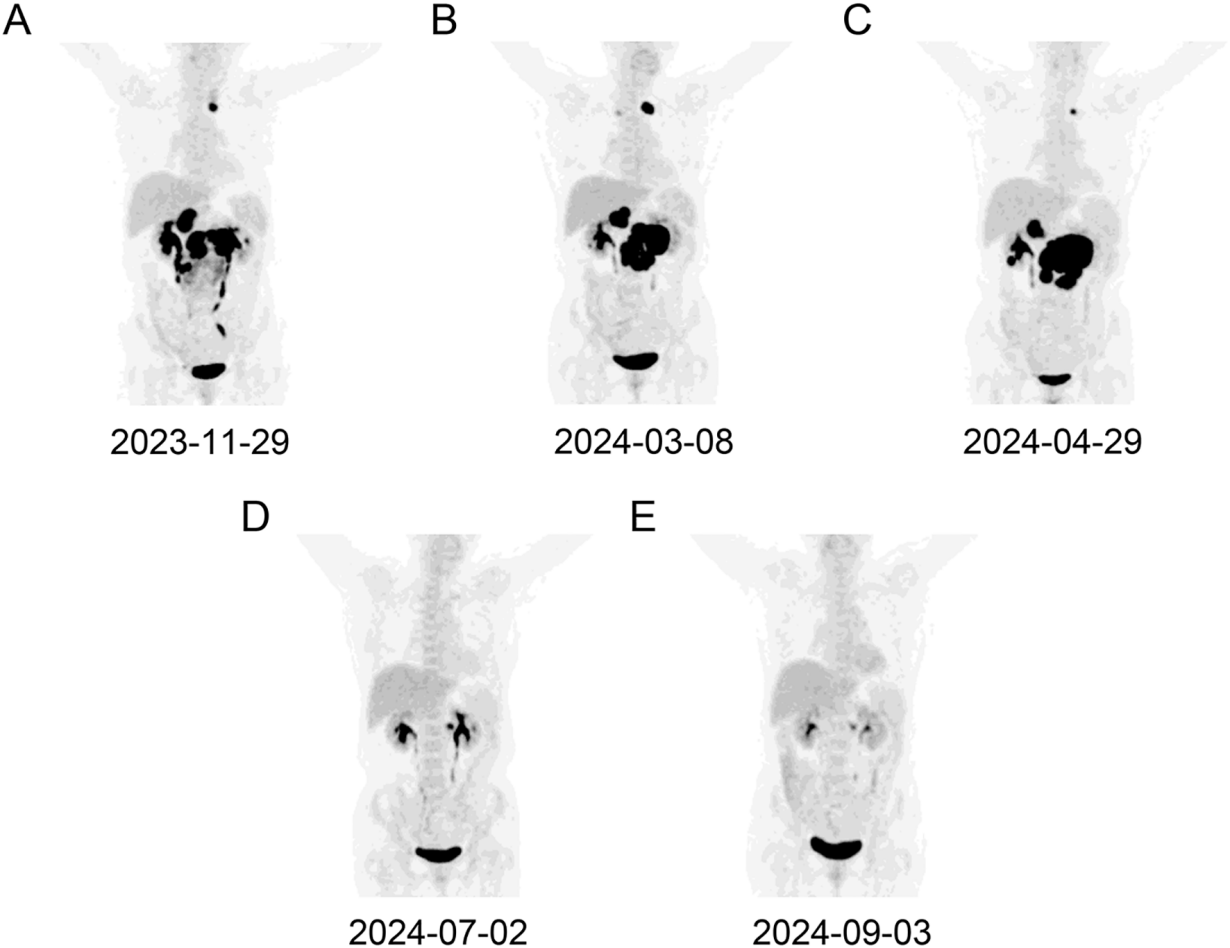
A dynamic evaluation of enlarged lymph nodes with PET-CT. The enlargement and reduction of lymph nodes at initial diagnosis **(A)**, PD following BR treatment **(B)**, PD following Pola-R-CHP treatment **(C)**, PR following glofitamab treatment **(D, E)**, respectively.

A 66-year-old Chinese woman was presented to our hospital on November 28, 2023, with a three-day history of abdominal pain. The patient had no significant medical history. No personal or family history of malignancies was documented. Psychosocial assessment revealed a retired factory worker living with spouse, with no history of smoking, alcohol use, or psychotropic medication. Physical examination showed that the bilateral supraclavicular lymph nodes were enlarged. Abdominal computed tomography (CT) scan showed multiple enlarged lymph nodes with partially fused, located around the abdominal cavity, along the retroperitoneal abdominal aorta, and adjacent to bilateral iliac arteries. Laboratory data showed that lactate dehydrogenase was elevated (760 U/L). Epstein-Barr virus-DNA test was positive (11200 copies/mL). Positron-emission tomography-computed tomography (PET-CT) showed high uptake of ^18^F-fluorodeoxyglucose in multiple lymphadenopathies distributed across the abdominal, retroperitoneum, left upper mediastinum, left cervical III and V regions, left peri-clavicular and bilateral diaphragmatic feet posterior regions (**Figure 1A**). Then, the abdomen lymph node biopsy was performed. Histopathological examination confirmed low-grade FL in the lymph nodes. The immunohistochemical (IHC) results were as follows: CD20 (+), Ki67 (30%+), CD10 (+), Bcl2 (+), CD3 (-), CK (-), CD56 (-), Cyclin D1 (-), CD5 (-), PAX5 (-), c-Myc (-), Bcl6 (-) and MUM-1 (-). The bone marrow (BM) examination did not reveal any lymphoma cells infiltration. The patient was diagnosed with FL (stage III, group A), classified as high risk by the FL International Prognostic Index [FLIPI (score 3)] and low risk by FLIPI-2 (score 1). Subsequently, the patient was treated with BR (rituximab 600mg day 0 and bendamustine 125 mg day 1-2) and continued this protocol for 3 cycles. No adverse events were observed in this treatment.

The patient was assessed in progressive disease (PD) after 3 cycles of BR therapy by PET-CT with a substantial increase in the size of the formed enlarged lymph nodes (**Figure 1B**). Consequently, a second abdominal lymph node biopsy was conducted. The lymph nodes pathology was high-grade B-cell lymphoma with a tendency toward DLBCL originating within germinal center B-cell-like (GCB). The IHC results were as follows: CD20 (diffuse +), CD3 (scatter +), Ki67 (60%+), CD10 (+), Bcl2 (+), Bcl6 (+), CD30 (-), CD5 (-), MUM-1 (-), Cyclin D1 (-) and c-Myc (-), and. BM examination revealed no lymphoma cells infiltration. The patient was diagnosed with DLBCL/tFL (GCB, stage III, group A), classified as high intermediate risk by the National Cancer Institute-International Prognostic Index (score 4), and low intermediate risk by Central Nervous System-International Prognostic Index (score 3). The patient was subsequently treated with Pola-R-CHP (rituximab 600mg day 0, polatuzumab vedotin 90mg day 1, cyclophosphamide 1000mg day 1, epirubicin 90mg day 1 and prednisone 85 mg day 1-5). On day 10 following the first cycle of Pola-R-CHP therapy, the patient developed grade 4 neutropenia (Common Terminology Criteria for Adverse Events v5.0), which resolved promptly with granulocyte colony-stimulating factor support.

**Figure 2:**
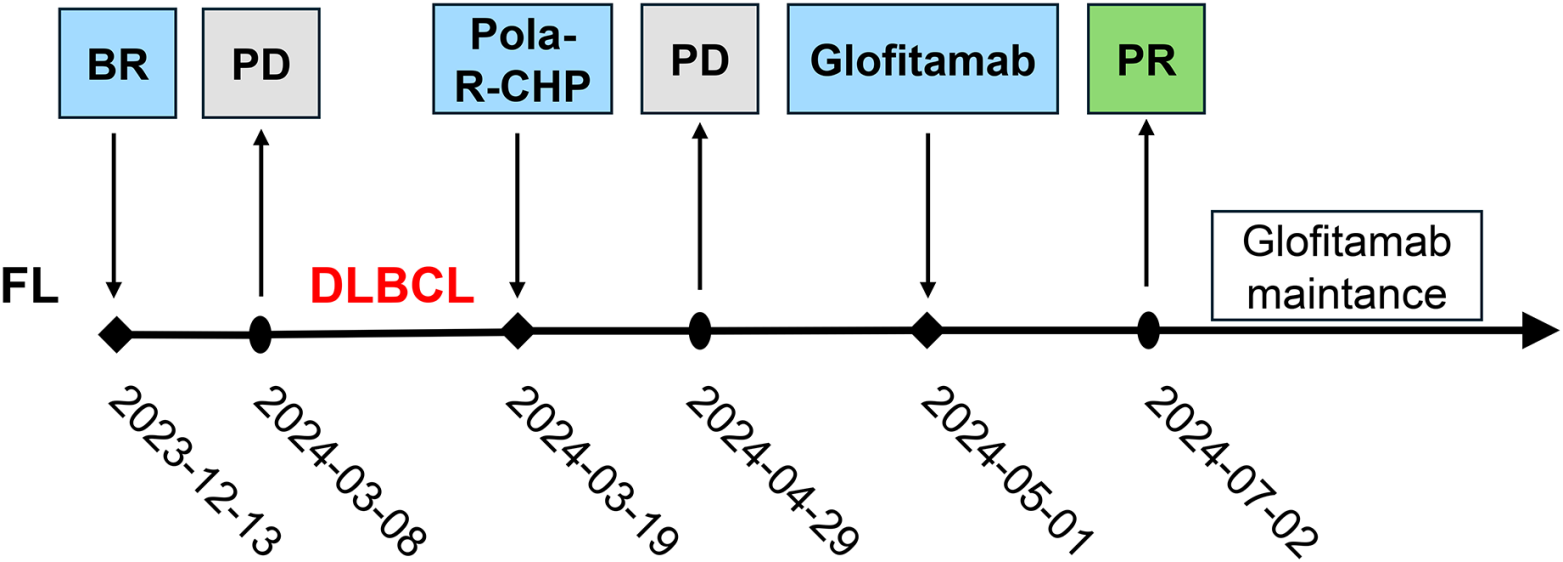
The timeline of treatment process in this case. FL, Follicular lymphoma; BR, bendamustine and rituximab; PD, progressive disease; DLBCL, Diffuse large B-cell lymphoma; Pola-R-CHP, polatuzumab vedotin, rituximab, cyclophosphamide, doxorubicin, and prednisone; PD, progressive disease; PR, partial response.

Unfortunately, the patient was assessed in PD after 2 cycles of Pola-R-CHP by PET-CT (**Figure 1C**). Subsequent cell-free DNA detection revealed that the molecular subtype was LymphGen-EZB/MYC^+^ with *EZH2*, *TNFRSF14*, *ETV6*, *SOCS1*, *BCL2* and *MYC* mutations. The fluorescence *in situ* hybridization revealed *MYC* and *BCL2* rearrangements without *BCL6* translocation, thus leading to the diagnosis of DLBCL/HGBL-*MYC*/*BCL2*. Given the primary refractory status of this patient to first-line therapy and the double-hit (DHIT) of *MYC* and *BCL2* rearrangements, the prognosis was considered adverse, and salvage treatment was only possible if a new treatment scheme was adopted. After a thorough deliberation, the CD20×CD3 bispecific antibody glofitamab was administered in a step-up dosage regimen, with 2.5 mg on day 8 and 10 mg on day 15 (cycle 1) followed by a 30 mg flat dose on day 1 (cycle 2-12) with each cycle spanning 21 days. The patient developed only grade 1 cytokine release syndrome (CRS) during cycle 1, which resolved with symptomatic management. No other significant adverse events (e.g., neurotoxicity, prolonged cytopenia) or unanticipated complications were observed. A partial response (PR) was observed on PET-CT evaluation after 3 and 6 cycles of glofitamab treatment (**Figure 1D, E**). In order to enhance the effect of glofitamab, the immunomodulatory agent lenalidomide was included in the treatment. Subsequently, the patient received glofitamab in combination with lenalidomide maintenance therapy until December 6th, 2024. The most recent CT scan revealed that the enlarged lymph nodes had ongoing shrunk after eight cycles of glofitamab therapy. Following glofitamab therapy, the median Functional Assessment of Cancer Therapy-Lymphoma (FACT-Lym) total score significantly increased from 93 [Interquartile Range (IQR): 86-110] to 140 (IQR: 131-155) at 6-month follow-up. The timeline of therapy is shown in **Figure 2**.

## Discussion

The available treatment options for relapse/refractory (R/R) DLBCL include second-line immunochemotherapy without cross-resistance, targeted therapy, immunotherapy, autologous hematopoietic stem cell transplantation and CAR-T (9, 11). However, the clinical outcome of R/R DLBCL remains unsatisfactory, particularly for those who are refractory to frontline treatment with an objective response rate of merely 20% and 1-year survival rate of only 29% (14). Herein, we present an elderly DLBCL patient who experienced transformation from FL, accompanied by DHIT, *MYC* and *BCL2* mutations, and LymphGen-EZB/MYC^+^ subtype. Transformed FL (tFL) has been observed to exhibit worse clinical outcomes, particularly in cases of histological transformation following frontline treatments (15–17). However, there is an absence of a consensus regarding therapeutic regimens for primary refractory DLBCL/HGBL-*MYC*/*BCL2* (18).

A paucity of prospective trials has been reported for the treatment of DHIT patients, of which extant reports on such patients are predominantly constituted by retrospective analyses or empirical treatments. Currently, precision and targeted therapy is a promising strategy to delay and overcome treatment resistance. For R/R DLBCL, current developments are focused on the utilization of CAR-T cell treatment and bispecific antibodies (19). CAR-T cell treatment has been demonstrated to be efficacious in R/R DLBCL with durable remission in 30%-40% (19). However, hindrance of CAR-T broader application is its intricate manufacturing process with a minimum of 3-4 weeks of production time and a high cost. Actually, these R/R patients exhibit rapid clinical progression and necessitate more expeditious treatment. Bispecific antibodies offer a distinct advantage in this regard, as they are readily available. To date, two bispecific antibodies, epcoritamab and glofitamab, have been granted approval by the Food and Drug Administration for use in DLBCL patients who have received ≥3 prior lines of therapy (20, 21).

A recent phase III controlled clinical trial confirmed a superior efficacy of the combination with glofitamab in R/R DLBCL (22). However, it should be noted that the tFL and DHIT patients were excluded from this clinical trial. Several retrospective clinical analyses have revealed that glofitamab improves the prognosis of R/R DLBCL, including tFL and DHIT patients (23–26). However, these analyses were conducted on small clinical cohorts, with even fewer cases of tFL and DHIT. We hereby present a complex case with multiple adverse events in addition to DHIT, as well as primary resistance to front-line intensive chemoimmunotherapy. In the present report, we applied glofitamab to an elderly patient with refractory DLBCL/HGBL-*MYC*/*BCL2*. The patient exhibited PR following 3 cycles of glofitamab treatment and was subsequently treated with glofitamab for a period exceeding 7 months. Currently, the patient is assessed as maintain the PR and continues to benefit from glofitamab therapy. The incorporation of lenalidomide into the Glofitamab formulation may have facilitated disease management through its immunomodulatory properties, encompassing heightened T-cell activation and a synergistic effect with bispecific antibodies. The present case demonstrates the efficacy of glofitamab in DLBCL/HGBL-*MYC*/*BCL2*, thereby establishing a foundation for subsequent studies in this field. Nevertheless, further clinical trials with larger sample sizes are required to ascertain the efficacy of these bispecific antibodies in these specific subtypes of DLBCL/HGBL patients. Such trials should address the diagnostic challenges inherent to this entity, which require integration of histopathology with molecular techniques (e.g., Fluorescence *in situ* hybridization for *MYC*/*BCL2* rearrangements or next-generation sequencing) to avoid misclassification. To ensure meaningful results, study designs should prioritize multicenter collaboration to overcome recruitment barriers and incorporate adaptive trial frameworks with biomarker-driven stratification. Potential feasibility challenges include centralized molecular profiling to confirm eligibility, management of bispecific antibodies related CRS in high-risk populations, and long-term follow-up to assess delayed neurotoxicity. Addressing these considerations will be essential to translate targeted immunotherapies into clinically actionable strategies for this molecularly defined subgroup.

In this case report, a patient with primary refractory DLBCL/HGBL-MYC/BCL2 achieved PFS of over 7 months (ongoing) with glofitamab, exceeding the median OS of 6.3 months reported in the international SCHOLAR-1 study (11). Notably, this response aligns with the subset of patients in Hsu et al. (60% 1-year PFS in responders) and Shumilov et al. (19% with sustained complete remission at 6 months) (24, 25), demonstrating durable benefits in aggressive, heavily pretreated disease. The outcome highlights the potential of glofitamab to induce prolonged disease control even after multiple prior therapies, including CAR-T and bendamustine-based regimens.

## Conclusion

In conclusion, we successfully treated a patient with transformed primary refractory DLBCL/HGBL-*MYC*/*BCL2* using the CD20×CD3 bispecific antibody glofitamab. Although only one successful case is presented, glofitamab could be considered as salvage therapy for transformed R/R DLBCL/HGBL-*MYC*/*BCL2*.

## Data Availability

The original contributions presented in the study are included in the article/Supplementary Material. Further inquiries can be directed to the corresponding author.
